# Effector, Memory, and Dysfunctional CD8^+^ T Cell Fates in the Antitumor Immune Response

**DOI:** 10.1155/2016/8941260

**Published:** 2016-05-22

**Authors:** John Reiser, Arnob Banerjee

**Affiliations:** University of Maryland School of Medicine, 20 Penn Street, Building HSFII, Lab No. S109, Baltimore, MD 21230, USA

## Abstract

The adaptive immune system plays a pivotal role in the host's ability to mount an effective, antigen-specific immune response against tumors. CD8^+^ tumor-infiltrating lymphocytes (TILs) mediate tumor rejection through recognition of tumor antigens and direct killing of transformed cells. In growing tumors, TILs are often functionally impaired as a result of interaction with, or signals from, transformed cells and the tumor microenvironment. These interactions and signals can lead to transcriptional, functional, and phenotypic changes in TILs that diminish the host's ability to eradicate the tumor. In addition to effector and memory CD8^+^ T cells, populations described as exhausted, anergic, senescent, and regulatory CD8^+^ T cells have been observed in clinical and basic studies of antitumor immune responses. In the context of antitumor immunity, these CD8^+^ T cell subsets remain poorly characterized in terms of fate-specific biomarkers and transcription factor profiles. Here we discuss the current characterization of CD8^+^ T cell fates in antitumor immune responses and discuss recent insights into how signals in the tumor microenvironment influence TIL transcriptional networks to promote CD8^+^ T cell dysfunction.

## 1. Introduction

Decades of research have resulted in substantial insights into the role of the adaptive immune system, including CD8^+^ T cells, in antitumor responses. In 1977, Fortner and Kripke demonstrated that tumor-challenged lymphocytes from irradiated donor mice were unreactive against syngeneic UV-induced tumors* in vitro* whereas tumor-challenged lymphocytes from nonirradiated mice rejected the same tumor. This finding implied that irradiation induced dysfunction of tumor-specific lymphocytes, which failed to reject the tumor [1]. In the mid-1980s, Rosenberg and colleagues defined tumor-infiltrating lymphocytes (TILs) as a subset of highly cytotoxic lymphocytes isolated from tumor-bearing patients that exhibited objective responses following adoptive transfer in human cancer patients [2, 3]. Further studies in athymic nude and SCID mice revealed that T cell deficiency correlates with a higher frequency of both spontaneous and chemically induced cancer, indicating a role for T cells in cancer immunosurveillance [4, 5]. In a study by Shankaran et al., the authors concluded that both lymphocytes and IFN*γ* were critical in antitumor immunity, suggesting a critical role for CD8^+^ T cells in antitumor immune responses [6]. Shortly after, Dudley et al. showed that a clonal repopulation of CD8^+^ TILs was responsible for tumor regression in patients with metastatic melanoma following lymphodepletion [7]. These studies highlighted a major role for CD8^+^ TILs in antitumor immune responses, supporting the use of tumor-specific CD8^+^ T cells in adoptive immunotherapy.

Clinical studies have shown a positive correlation between the frequency of CD8^+^ TILs and cancer-free survival in patients with breast, lung, melanoma, colorectal, and brain cancer [8–12]. Current immunotherapies involve enhancing the activity of antigen-specific CD8^+^ TILs through cytokine treatment, immune checkpoint blockade, chimeric antigen receptor therapy, and adoptive T cell transfer (ACT) [13]. Despite some clinical success, ACT experiments in both humans and mice have shown that initial tumor regression often yields to uncontrolled relapse [14, 15]. This suggests that the initial T cell response incompletely eliminates tumor cells and that, upon regrowth, tumor-specific T cells become unable to control the tumor. This finding has been supported in human patients as analysis of tumor-infiltrated lymph nodes (TILN) in late-stage melanoma patients revealed an aberrant tumor-specific T cell phenotype as compared to the phenotype observed in circulating effector, memory, and naïve T cells [16]. A separate study in late-stage melanoma patients found that a fraction of circulating antigen-specific CD8^+^ T cells are functionally impaired, supporting the coexistence of multiple T cell fates in the antitumor immune response [17].

There is no universally accepted classification system of CD8^+^ T cell fates in the context of antitumor immunity. Classifying CD8^+^ T cell subsets is challenging due to lack of fate-specific biomarkers, unclear subset distinction, and disparity between cancer types. However, at least six subsets of CD8^+^ T cell fates have been defined in both cancer patients and experimental models. These include effector T cells, memory T cells, exhausted T cells, anergic T cells, regulatory T cells, and senescent T cells. The following sections highlight the current view of CD8^+^ T cell fates in the context of the antitumor immune response, including the transcriptional regulation of cell fate determination.

## 2. Characterization of CD8^**+**^ T Cell Fate in the Antitumor Immune Response

### 2.1. Effector CD8^+^ T Cells

**Figure 1:**
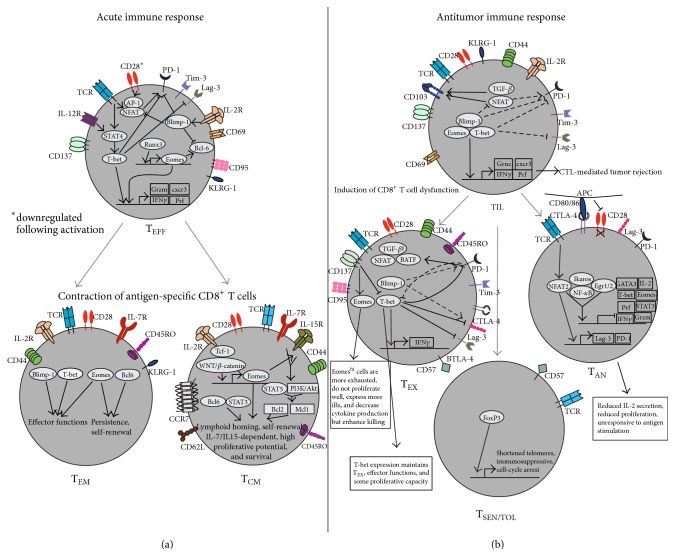
Characterization of CD8^+^ T cell fates in acute and antitumor immune responses. (a) In an acute immune response, CD8^+^ T cell priming induces cytotoxic T_EFF_ regulated by the transcription factors T-bet, Runx3, Eomes, Blimp-1, and NFAT and the cytokines IL-2 and IL-12. Following antigen clearance, T_EFF_ contract into T_EM_ and T_CM_. T_EM_ are regulated by different levels of T-bet/Eomes and Blimp-1/Bcl-6. T_CM_ have higher levels of Eomes and Bcl-6 as compared to T_EM_ and are influenced by expression of Tcf-1, WNT/*β*-catenin, STAT3, and STAT5, which cooperate to maintain a persistent population of T_CM_ with high proliferative potential. IL-7 and IL-15 maintain homeostatic proliferation of CD8^+^ memory T cells. (b) Tumor antigen primed T_EFF_ traffic to tumors as TILs. T-bet and Blimp-1 cooperate to repress iR expression and, with Eomes, promote CTL-mediated tumor rejection. NFAT and TGF-*β* promote tumor cell lysis through CD103 expression. Dysfunctional TIL can become T_EX_, T_AN_, or T_SEN/TOL_. High T-bet expression maintains functional T_EX_ whereas high Eomes expression promotes severe exhaustion. There is complex interplay between T-bet, Blimp-1, and iRs in T_EX_. T_AN_ result from insufficient costimulation through CD28. Unbalanced NFAT signaling induces anergy-inducing genes and, along with Ikaros, Egr1/2, and NF-*κ*B, inhibits effector molecule expression. T_SEN/TOL_ lack CD28 expression and may be regulated by FoxP3.

Naïve CD8^+^ T cells differentiate into effector T cells (T_EFF_) upon TCR engagement with antigen and costimulation by an antigen-presenting cell (APC). In antitumor responses, robust CD8^+^ T cell priming occurs primarily in tumor-draining lymph nodes (TDLNs). Activation and differentiation of effector CD8^+^ T cells can also occur directly in the tumor by tissue-resident, cross-presenting APCs as well as tumor cells themselves [45–48]. T_EFF_ are identified based on the expression of surface markers such as CD25, CD69, CD95, CD137, and KLRG-1 [18–20] (Table 1 and Figure 1). Terminally differentiated T_EFF_ are IL-2 dependent and highly cytotoxic, rapidly expressing high levels of IFN*γ*, TNF*α*, perforin, and granzymes following activation [21, 22]. Tumor antigen-specific T_EFF_ that efficiently invade primary tumor lesions are termed TILs. TILs recognize and lyse tumor cells both* in vitro* and* in vivo*; however* in vivo* antitumor T cell responses are variable, owing to disparity in T cell activation, cytokine signaling, and immunosuppressive mechanisms between tumor types [49–52]. T_EFF_ likely represent the majority of the TIL population in well-controlled tumors and are responsible for positive clinical responses, as adoptive transfer experiments using autologous T_EFF_ derived from CD8^+^ TILs successfully eradicate tumors in cancer patients [3, 7, 9, 53, 54]. In acute immune responses, T_EFF_ are short-lived and undergo apoptosis upon elimination of antigen [55]. However, tumor load or prime-boost cancer vaccines can chronically stimulate CD8^+^ T cells, leading to phenotypic changes and functional impairment. The switch from a highly active CD8^+^ TIL population to chronically stimulated CD8^+^ T cells favors the tumor over the host immune response and can ultimately lead to immune escape (Figure 1) [56]. The dysfunctional CD8^+^ T cell fates that are induced by uncontrolled tumor load are discussed in detail below.

### 2.2. Memory CD8^+^ T Cells

In several types of acute infectious challenges, T_EFF_ undergo a rapid, apoptosis-induced contractile phase following antigen clearance. After resolution of acute infection, a small subset of antigen-experienced CD8^+^ T cells remains as memory CD8^+^ T cells [57–60]. It should be noted that adaptive “memory” implies the absence of antigen, a condition that is not often met in an antitumor immune response. For the remainder of this discussion, we will continue to refer to these cells as memory CD8^+^ T cells, though they may more appropriately be characterized as “persistent” CD8^+^ T cells in the context of the antitumor immune response.

Memory CD8^+^ T cells were subdivided in 1999 into two broad subsets, central memory (T_CM_) and effector memory (T_EM_), distinguished by the relative expression of two homing molecules, CD62L and CCR7 [23–25]. T_EM_ have a phenotype more similar to that of effector cells, characterized by a loss of CCR7 expression and intermediate to no CD62L expression. These cells exhibit rapid effector function, readily differentiating into T_EFF_ that secrete high amounts of IFN*γ* and are highly cytotoxic [26]. In contrast, T_CM_ are less differentiated, have increased proliferative potential and greater self-renewal capability, can produce high amounts of IL-2, and acquire effector functions less rapidly. Upon secondary antigen challenge, both subsets give rise to progeny that differentiate into T_EFF_ [27, 28, 56]. Subsets of tumor-specific T_EM_ and T_CM_ have been identified in breast and colorectal cancer patients [61–64]. Similarly, studies in both mice and humans have demonstrated that memory CD8^+^ T cells develop* in vivo* following adoptive transfer, maintain effector capabilities, and mediate tumor regression [65, 66].

### 2.3. Exhausted CD8^+^ T Cells

Exhausted T cells (T_EX_) are defined as a persistent T cell population with low IL-2 and IFN*γ* production, reduced cytotoxic activity, reduced proliferative potential, and eventual deletion of the population of antigen-specific T cells [29, 75]. T cell exhaustion is observed in the context of uncontrolled viral infection and cancer, and investigators believe that chronic antigen exposure drives CD8^+^ T cells to an exhausted fate [29, 75, 76]. A number of inhibitory receptors (iRs) are upregulated on T_EX_, indicating a role for these receptors in the attenuation of T cell function. In healthy individuals, iRs on CD8^+^ T cells promote self-tolerance and prevent autoimmunity by competing for costimulatory receptor ligands, attenuating positive TCR signaling, and/or inducing immunosuppressive genes. In the context of an antitumor immune response, elevated expression of multiple iRs promotes CD8^+^ T cell exhaustion and immune evasion. Some of these receptors include PD-1, CTLA-4, TIM-3, LAG-3, CD160, BTLA, TIGIT, and 2B4 [29–33] (Table 1).

Early experimental evidence for CD8^+^ T cell exhaustion in antitumor immunity was observed in a transgenic mastocytoma cell line overexpressing programmed death-ligand 1 (PD-L1). This cell line resisted TCR-mediated cell lysis* in vitro* and was more tumorigenic and invasive* in vivo* [77]. In the same year, Dong et al. demonstrated that PD-L1 was expressed in lung, ovary, and colon cancers as well as melanomas [78]. Further studies revealed that T_EX_ expressed high levels of PD-1 in Hodgkin's lymphoma, melanoma, hepatocellular carcinoma, and gastric cancer patients [79–82]. CTLA-4 is another T cell-specific iR known to be upregulated in exhausted T cells [31]. In the early 2000s, investigators began testing anti-CTLA-4 antibodies for their ability to reverse T cell dysfunction in cancer patients. In 2011, Ipilimumab became the first FDA-approved immune checkpoint inhibitor, approved for the use in patients with late-stage metastatic melanoma [83, 84]. A more recent study showed that dual blockade of CTLA-4 and PD-1 corresponded with reversal of T cell exhaustion, characterized by increase cytokine release, suppression of Tregs, and upregulation of signaling molecules associated with activation. Dual blockade led to tumor rejection in murine models of ovarian and colon carcinoma [85]. An elegant study from Baitsch et al. revealed a marked distinction between CD8^+^ T cell fates in patients with metastatic melanoma. While circulating tumor-specific T cells exhibited normal effector function, TILs isolated from the tumor-draining lymph node (TDLN) showed a markedly exhausted phenotype, characterized by decreased IFN*γ* expression and upregulation of CTLA-4 and Lag-3. The investigators concluded that T_EFF_ and T_EX_ coexist in patients with metastatic melanoma, supporting the coexistence of multiple CD8^+^ T cell fates in antitumor immune responses. The study further highlights the complexity of the tumor microenvironment as a largely immunosuppressive environment and suggests that tumor-specific expression of ligands for T cell iRs promotes immune evasion [16]. The discovery that T cell exhaustion could be reversed* in vitro* (removal from immunosuppressive environment) and* in vivo* (immune checkpoint blockade) has prompted the rapid development of other immune checkpoint inhibitors as novel immunotherapies [86, 87]. Detailed reviews of FDA-approved and clinical trial immune checkpoint inhibitors have been described elsewhere [88–90].

Though iRs are classically used to identify T_EX_ *in vivo*, many of these receptors are upregulated following T cell activation. Legat et al. showed that PD-1, CTLA-4, and LAG-3 were upregulated upon T cell activation in the antitumor response. In contrast to the study by Baitsch et al., the authors demonstrate that CD8^+^ T cells isolated from both metastatic and nonmetastatic lymph nodes in melanoma patients exhibit increased expression of iRs and decreased cytokine production [91]. PD-1 expression was found to identify patient-specific, tumor-reactive TILs in a number of human tumors. Expression of the iRs PD-1, LAG-3, and TIM-3 correlated with antigen-experienced CD8^+^ TILs that recognized and lysed autologous tumor cell lines [92]. In line with this idea, Duraiswamy et al. showed that CD8^+^PD-1^hi^ T cells from healthy donors exhibit a distinct transcriptional profile as compared to CD8^+^PD-1^hi^ T cells in HIV-infected patients. In healthy donors, PD-1 expression correlated with a T_EM_ phenotype as opposed to terminal T_EFF_ [93]. Thus, canonical identification of T_EX_ by iR expression does not always correlate with T cell dysfunction. The correlation of iR expression and CD8^+^ T cell exhaustion needs to be further investigated and may depend on activation state, quantity of expression, coexpression of multiple receptors, and strength of the inhibitory signal. Indeed, studies have shown that iR expression and signal strength influence CD8^+^ T cell fate towards an exhausted phenotype in infectious disease and cancer [94, 95].

CD8^+^ T cell exhaustion represents a distinct but reversible T cell fate in the context of antitumor immune responses. At least some iRs are expressed on activated T_EFF_, and it remains incompletely defined to what extent individual iRs contribute to the functional impairment of CD8^+^ T cells observed in cancer as opposed to serving as phenotypic markers of exhaustion. Anticancer activity of iR-blocking antibodies in mice and humans supports at least a partial direct role for these receptors in T cell dysfunction [89, 96]. Continued work in this area will help determine which iRs best identify exhausted T cells and are most amenable to therapeutic targeting. Similarly, further insight into iR signaling may allow targeting of specific downstream molecules.

### 2.4. Anergic CD8^+^ T Cells

One of the pivotal obstacles in immunotherapy is overcoming tolerance. Central tolerance deletes self-reactive T cells with high avidity TCRs for self-antigen. Self-antigen-specific T cells that escape the thymus are often tolerized in the periphery, through either deletion or induction of anergy [97]. Because tumor antigens are often nonmutated self-antigens, these two processes significantly impair the host's ability to mount an effective antitumor immune response [98]. Anergy refers to a hyporesponsive state of impaired IL-2 production and proliferation, resulting from inefficient costimulation and/or high coinhibitory signaling or from partial or chronic TCR stimulation [99]. In antitumor immune responses, the scarcity of circulating tumor-specific T cells, most of which express low-avidity TCRs, impedes the recognition and destruction of tumor cells [49]. Nevertheless, tumor antigen-specific TILs can be found at high numbers in many cancer types, though often unable to control the tumor [100]. Anergy is usually characterized* in vitro*, but anergy induction* in vivo* promotes what is referred to as T cell tolerance [101]. It is difficult to accurately identify anergic/tolerant T cells (T_AN_ and T_TOL_, resp.) in* in vivo* cancer models due to a lack of distinctive biomarkers. However, multiple studies suggest that immunosuppressive mechanisms in the tumor microenvironment are capable of promoting an anergic phenotype. Cancer cells and tumor-associated APCs can express high levels of coinhibitory molecules, and both APCs and cancer cells directly activate CD8^+^ T cells* in vivo* [47, 102]. A combination of CD8^+^ T cells priming with strong coinhibitory signaling might promote T cell anergy in the tumor microenvironment. Studies have validated expression of B7 family members on myeloid dendritic cells, tumor-associated macrophages, and cancer cells. These studies also showed that blockade of inhibitory B7 molecules reduced tumor growth* in vivo* [102–104].

The transcriptional network that promotes CD8^+^ T cell anergy is complex and many of the transcription factors that promote an anergic phenotype also promote T cell exhaustion. Still, evidence suggests that these two CD8^+^ T cell fates are distinct in antitumor immune responses [99, 105]. In a model of chronic LCMV infection, it was shown that gene expression profiles from CD8^+^  T_AN_ and T_EX_ were significantly different, suggesting a functional difference between the two subsets (see below) [106]. From a temporal standpoint, the development of T cell anergy is believed to occur before or in the early stages of tumor progression. For one, anergy induction in the thymus or periphery renders it unlikely that a significant number of tumor-reactive CD8^+^ T cells exist in circulation even before a tumor is established [97]. Along these lines, a study by Staveley-O'Carroll et al. suggests that T cells are rendered anergic in the early stages of tumor progression [107]. On the other hand, CD8^+^ T cell exhaustion is an eventual state of T cell dysfunction that occurs in progressive stages and varies depending on the context and abundance of antigen [29, 31, 105]. Collectively, these studies imply that CD8^+^ T cell anergy occurs before or in the early stages of tumorigenesis whereas exhaustion is a gradual state of T cell dysfunction. Further analysis of dysfunctional CD8^+^ T cells in multiple stages of tumor development and different tumor types will help further delineate the role of T_AN_ and T_EX_ in antitumor immune responses.

### 2.5. Senescent/Regulatory CD8^+^ T Cells

Senescent T cells (T_SEN_) are defined by loss of CD28 expression, permanent cell-cycle arrest, and shortened telomere length. It is well known that T_SEN_ have implications in human ageing, but their role in cancer is less clear [42]. Interestingly, CD8^+^ T cells displaying a senescent phenotype (CD8^+^CD28^−^) have been associated with suppressor function* in vitro* [108, 109], indicating a potential immunosuppressive role in antitumor immune responses. Similarly, populations of regulatory CD8^+^ T cells have been identified in head and neck and lung cancer, marked by lack of CD28 expression [43, 110]. Thus, CD8^+^CD28^−^ T cells may comprise a heterogeneous population, containing both senescent and/or regulatory CD8^+^ T cells. A comprehensive study by Filaci et al. revealed that CD8^+^CD28^−^ regulatory T cells (T_REG_) are present in metastatic lymph nodes in a number of cancers. This study concluded that CD8^+^CD28^−^  T_REG_ reduced T_EFF_ proliferation and cytolytic capacity via IL-10 secretion [111]. However, this study did not identify this population of CD8^+^CD28^−^ cells as senescent, but instead as a regulatory T cell population, similar to but phenotypically distinct from CD4^+^FoxP3^+^  T_REG_. Thus CD28 expression alone may not distinguish between CD8^+^  T_SEN_ and T_REG_. Montes et al. demonstrated that tumor cell lines could induce properties characteristic of CD8^+^CD28^−^ regulatory/senescent T cells, including shortened telomeres and immunosuppressive activity. Importantly, the study showed that inhibition of T_EFF_ proliferation was contact-dependent [112]. The same group then demonstrated that CD8^+^CD27^−^CD28^−^  T_SEN_ could similarly be induced by soluble factors and that this phenotype is inhibited by exogenous IL-7 [113]. It remains to be determined whether these populations represent distinct T cell fates or comprise a single CD8^+^ T cell subset and how the context of tumor control and tumor type contribute to the differentiation/maintenance of CD8^+^  T_SEN_ and T_REG_. One study demonstrated that CD8^+^CD28^−^ expression identifies a T cell subset that recognizes and responds to HPV-induced cervical cancer, suggesting that CD28 may not serve as a reliable biomarker for CD8^+^  T_SEN_/T_REG_ [114]. In line with this idea, CD57 was found to be a marker of replicative senescent T cells in a model of HIV infection, regardless of CD28 expression [44]. A recent study illuminated the impact of senescent CD8^+^ T cells in patients with late-stage lung cancer. The CD8^+^ T cell population in patients was consistent with an immunosenescent phenotype, based on CD28 and CD57 expression, before the onset of chemotherapy. Following chemotherapy, the proportion of senescent and terminally differentiated CD8^+^CD28^−^CD57^+^ cells was significantly increased in stage IV lung cancer patients as compared to the healthy controls. Similarly, the population of naïve and memory CD8^+^CD28^+^CD57^−^ T cells was decreased in the same patients as compared to healthy controls. These findings suggest that the number of CD8^+^CD28^−^CD57^+^  T_SEN_ cells correlates with disease stage in late-stage lung cancer patients, offering a role for CD8^+^  T_SEN_ in antitumor immune responses [115]. Further phenotypic and functional analysis of CD8^+^  T_SEN_ and T_REG_ is needed to characterize these cells as individual CD8^+^ T cell fates.

## 3. Which Subset Promotes Optimal Antitumor Immune Responses?

There is conflicting evidence as to which subset of CD8^+^ T cell promotes superior antitumor immunity. Adoptive T cell transfer of T_EFF_ promotes robust responses, but these cells often exhibit reduced persistence* in vivo* [7, 9, 54]. Initial antitumor responses often yield to tumor recurrence and the population of antigen-specific T cells becomes functionally impaired [15]. Gattinoni et al. found that more differentiated T_EFF_ were increasingly cytotoxic* in vitro* but exhibited impaired proliferative capacity and antitumor activity* in vivo* [67]. Still, multiple studies have shown that transfer of highly active T_EFF_ leads to tumor rejection in both humans and mice [3, 52, 53, 68]. One study showed that terminal T_EFF_ cultured* in vitro* transitioned into a smaller population of T_EM_ that promoted tumor regression and persisted for 2 months after transfer in patients with metastatic melanoma [69]. Both T_CM_ and T_EM_ from human breast cancer patients selectively homed to and rejected tumors in NOD/SCID mice with breast cancer, suggesting that both memory subsets can promote antitumor activity* in vivo* [61]. In a murine model of melanoma,* in vitro*-generated T_CM_ exhibited robust expansion and rejected tumors* in vivo* whereas T_EM_ did not [70]. Wu et al. demonstrated that TCR-transgenic T_CM_ displayed both an effector and memory phenotype and possessed superior antitumor activity as compared to TCR-transgenic T_EFF_ [71]. These studies suggest increased efficacy of less-differentiated T_CM_ in adoptive cell transfer therapy. The ability to promote the development of functional T cell memory* in vitro* and* in vivo* may provide a mechanism to enhance CD8^+^ T cell-mediated antitumor immune responses.

More recently, two additional subsets of memory T cells have been identified: tissue-resident memory T cells (T_RM_) and T memory stem cells (T_SCM_) [8, 57, 72–74]. Djenidi et al. identified a subset of TILs that correlated with increased survival in patients with non-small-cell lung carcinoma (NSCLC). The authors characterized these cells as T_RM_ based on surface expression of CD8, CD103, PD-1, and Tim-3 [8]. T_RM_ are a relatively recently defined subset, and it remains to be determined to what degree these cells represent a distinct memory T cell subset, although emerging evidence suggests that they are transcriptionally, phenotypically, and functionally different from T_EM_ and T_CM_ [74]. Gattinoni et al. characterized a population of T_SCM_ based on expression of surface markers distinctive of both naïve (CD45RO^−^, CCR7^+^, CD45RA^+^, CD62L^+^, CD27^+^, CD28^+^, and IL-7R*α* ^+^) and memory (CD95^+^, IL-2R*β* ^+^, CXCR3^+^, and LFA-1^+^) CD8^+^ T cells. This cell population proliferated more efficiently and elicited better antitumor immune responses as compared to T_CM_, suggesting that the T_SCM_ population might yield more objective antitumor responses owing to its less-differentiated state and increased proliferative potential [72]. These studies offer a more complex view of T cell memory subsets, where multiple stages of memory T cell differentiation correlate with phenotypic and functional changes.

## 4. Transcriptional Regulation of CD8^+^ T Cell Fate Decision in Antitumor Immune Responses

It is well known that CD8^+^ T cell fate in the tumor microenvironment is influenced by multiple factors including the nature of antigen stimulation/CD8^+^ T cell priming, soluble and cell-surface immunomodulatory ligands, and nutrient and oxygen availability [116–119]. CD8^+^ T cell dysfunction is likely caused by a combination of immunosuppressive mechanisms. It is unclear how all of these factors regulate the transcriptional profile of dysfunctional CD8^+^ TILs. In this section, we will discuss transcriptional changes that promote the differentiation of different CD8^+^ T cell fates in antitumor immune responses.

### 4.1. Anergic/Tolerant CD8^+^ T Cells

Few studies have demonstrated that T_AN_ or T_TOL_ CD8^+^ T cells persist at a relevant level in cancer but it stands to reason that these cells could play a significant role in mediating immune evasion. Tolerant or anergic CD8^+^ T cells that would otherwise respond to a tumor-specific antigen (TSA) or tumor-associated antigen (TAA) would be unable to trigger an effective immune response against transformed cells.

Transcriptional networks in anergic CD4^+^ T cells have been studied both* in vitro* and* in vivo*. Strong TCR stimulation in the absence of sufficient costimulatory signaling via CD28 leads to activation of NFAT (nuclear factor of activated T cells) without activation of AP-1 (activator protein 1). The absence of NFAT/AP-1 heterodimerization allows NFAT homodimerization and promotes the expression of anergy-inducing genes including Egr2, Ikaros, and members of both the E2F transcription factors and the E3 ubiquitin ligase family. Many of these anergy-inducing genes then repress critical effector molecules including IL-2, IFN*γ*, and TNF*α* [99, 120, 121]. Few studies have attempted to elucidate the transcriptional network in CD8^+^  T_AN_ and the anergy-inducing genes that promote tolerance remain relatively uncharacterized. In an* in vivo* anergy induction model, Srinivasan and Frauwirth demonstrated a defect in calcium signaling in CD8^+^ T cells, which resulted in translocation of NFAT2 but not NFAT1 to the nucleus. This suggests a signaling network whereby NFAT isoforms become activated in response to different concentrations of intracellular calcium and NFAT2 regulates expression of anergy-inducing genes [34] (Figure 1). In primary culture, Ikaros haploinsufficient CD8^+^ T cells produced autocrine IL-2 and differentiated into IFN*γ*-secreting CTL without the addition of exogenous IL-2. These cells exhibited enhanced efficacy against B16 melanoma tumors* in vivo* as compared to WT cells, suggesting a role for Ikaros in maintaining tolerant CD8^+^ T cells [35]. Genetic ablation of the E3 ubiquitin ligase Cbl-b was shown to prevent induction of anergy in TCR-transgenic CD8^+^ T cells* in vivo* [36]. Similarly, blockade of the iR Lag-3 was shown to rescue tolerant CD8^+^ T cells in a self-tolerance and tumor model. Upon Lag-3 blockade, CD8^+^ T cells exhibited restored effector function and accumulated at greater numbers in tumor tissue [37]. In line with this idea, an intricate study by Schietinger et al. compared gene signature profiles between naïve, memory, tolerant, rescued, and retolerized CD8^+^ T cells. Lag-3 was found to be significantly upregulated in tolerant CD8^+^ T cells. Similar to CD4^+^ T cells, Egr1/Egr2 were downregulated in rescued and memory CD8^+^ T cells. Effector genes such as Infg, Prf1, and Gzmm were found to be upregulated in rescued and memory CD8^+^ T cells as were the transcription factors Tbx21, Eomes, Gata3, and Stat3 as well as multiple chemokine and cytokine molecules. Gene signature profiling also revealed significant differences in genes regulating chromatin modification in tolerant versus retolerized CD8^+^ T cells, implying that epigenetic changes are critical in CD8^+^ T cell fate decision [38].

One study implicated the iR PD-1 in the induction of CD8^+^ T cell anergy* in vivo* [39]. PD-1 is known to inhibit T cell function through different mechanisms, including negative signaling upon TCR engagement through phosphatase recruitment [122]. NFAT promotes PD-1 expression in early activated CD8^+^ T cells and unbalanced NFAT signaling may therefore contribute to T cell anergy through PD-1 expression [123] (Figure 1). Thus, interplay between transcription factors and iRs promotes various states of CD8^+^ T cell dysfunction including exhaustion and tolerance.

The NF-*κ*B transcription factor family is known to regulate T cell-specific gene expression and NF-*κ*B is necessary to mediate CD8^+^ T cell tumor rejection* in vivo* [40]. One study showed that T cells from tumor-bearing mice exhibited decreased IFN*γ* production that correlated with expression of distinct NF-*κ*B/Rel isoforms, suggesting that NF-*κ*B signaling influences T cell effector function in antitumor immune responses [41]. A recent study by Clavijo and Frauwirth supports these findings, as they found that T_AN_ exhibit impaired NF-*κ*B activation in a model of T cell tolerance [124]. Further studies are needed to facilitate accurate characterization of CD8^+^  T_AN_/T_TOL_ and elucidate their role in antitumor immune responses.

### 4.2. Senescent/Regulatory CD8^+^ T Cells

There is little known concerning the transcriptional networks involved in CD8^+^  T_SEN_/T_REG_ in the context of antitumor immune responses, yet studies suggest that tumors are capable of inducing a T_SEN_/T_REG_ phenotype both* in vitro* and* in vivo* [43, 110, 112]. CD8^+^CD28^−^  T_REG_ were found to express higher levels of FoxP3 mRNA in patients with lung cancer, suggesting the existence of a regulatory CD8^+^CD28^−^ population in cancer patients, possibly regulated by the expression of FoxP3 [43]. Similarly, two studies identified a CD8^+^FoxP3^+^ subset of T_REG_ in patients with colorectal and prostate cancer, suggesting that FoxP3 can be expressed in CD8^+^ T cells and promote an immunosuppressive phenotype in cancer patients [125, 126]. Another study highlighted similarities between CD8^+^Foxp3^+^ T cells and CD4^+^ Foxp3^+^ T cells in terms of phenotypic markers and lack of effector molecules but found that the CD8^+^ subset does not possess potent suppressive activity [127]. Currently, whether T_SEN_ and T_REG_ are two distinct T cell fates or represent a mutual phenotype remains to be determined. Ramello et al. offered a potential mechanism by which tumor-induced CD8^+^  T_SEN_ promote tumorigenesis by influencing monocyte and macrophage secretion of proinflammatory cytokines and angiogenic factors. CD8^+^  T_SEN_ increased monocyte/macrophage-specific production of IL-1*β*, TNF, and IL-6, MMP-9, VEGF-A, and IL-8 in a contact-dependent manner. Importantly, this proinflammatory phenotype was found to be dependent on Tim-3 and CD40L as blocking antibodies against these receptors reduced production of many of the proinflammatory factors [128]. This study does not identify transcription factors involved in CD8^+^  T_SEN_ signaling but implies that costimulatory/coinhibitory receptors play a role in promoting this fate. The authors did not characterize the suppressive activity of the CD8^+^  T_SEN_ on other T cells, and so it is unknown whether this subset of cells was functionally distinct from CD8^+^  T_REG_. The characterization of the transcription factors that regulate these phenotypes will help advance our understanding of the role of CD8^+^  T_SEN_/T_REG_ in antitumor immune responses.

### 4.3. Exhausted CD8^+^ T Cells

CD8^+^  T_EX_ represent the most commonly identified subset of dysfunctional T cells in antitumor immune responses. Expression of cell fate-influencing transcription factors in exhausted CD8^+^ T cells has been investigated in models of chronic viral infection to a greater degree than in cancer models. Though few studies have examined the transcriptional profile of CD8^+^  T_EX_ in cancer, crosstalk between iRs and transcription factors is indicated in promoting this fate. Persistent antigenic stimulation and inflammation are characteristics of both chronic viral infection and cancer, and, thus, transcriptional programming of exhaustion in the two disease states may be similar [16, 29].

Both T-bet and Eomes are known to be important in antitumor immune responses, consistent with their role as mediators of effector function in CD8^+^ T cells [129, 130]. T-bet expression was found to correlate with increased cancer-free survival in human colorectal cancer patients [10]. Studies in mice have identified multiple roles for T-bet and Eomes in antitumor immune responses, including controlling CD8^+^ T cell number, trafficking, effector function, and memory recall responses [129, 131]. One study demonstrated that exhausted CD8^+^ TILs express low levels of both T-bet and Eomes. PD-1, PD-L1, and CTLA-4 antagonism increased levels of both T-bet and Eomes and restored effector function [85]. Similarly, Berrien-Elliott et al. showed that blockade of CTLA-4, PD-1, and LAG-3 increased T-bet but not Eomes expression in CD8^+^ T cells. Reexpression of T-bet was required for IFN*γ* production and cytotoxic activity against FBL leukemia in mice [132]. This study suggests a feedback loop between T-bet and PD-1, as T-bet is known to repress PD-1 expression and maintain CD8^+^  T_EX_ in chronic infection [133]. Our lab has shown that T-bet and Eomes are coexpressed with iRs PD-1 and LAG-3 as well as costimulatory receptors 4-1BB and OX40 in exhausted CD8^+^ TILs in a murine lymphoma model. Agonistic ligation of 4-1BB was associated with increased Eomes, decreased T-bet expression, and delayed tumor growth [134]. One study found that T-bet expression was decreased in CD8^+^ T cells in a model of chronic LCMV infection. Overexpression of T-bet in P14 cells repressed PD-1, Lag-3, CD160, and BTLA [133]. In another study of chronic viral infection, CD8^+^  T_EX_ consisted of a majority of Eomes^hi^PD-1^hi^ population and a much smaller, but highly proliferative, T-bet^hi^PD-1^int^ population. This study suggests a dynamic conversion from T-bet^hi^ to Eomes^hi^ virus-specific CD8^+^ T cells during a state of persistent antigen challenge and that these two populations cooperate to control viral infection [135]. Buggert et al. compared T-bet and Eomes expression between patients with acute viral infection (CMV) and chronic viral infection (HIV). Similar to the previous studies, HIV patients displayed an exhaustive CD8^+^ T cell profile characterized by high Eomes expression and low T-bet expression. This population of cells displayed elevated expression of multiple iRs [136]. These studies imply a heterogeneic population of antigen-specific CD8^+^ T cells in chronic viral infection and cancer, where CD8^+^ T cells eventually display an exhaustive phenotype characterized by high Eomes and low T-bet expression. These studies suggest that T-bet and Eomes have distinct roles in CTL-mediated antitumor immune responses. Whereas T-bet promotes terminal differentiation in acute immune responses, it maintains effector functions in CD8^+^  T_EX_. On the other hand, high Eomes expression correlates with severe CD8^+^ T cell exhaustion. The above studies suggest complex interplay between iRs and T-bet and Eomes in exhausted CD8^+^ T cells and differential costimulatory/coinhibitory receptor signaling likely influences their expression as well as CD8^+^ T cell fate (Figure 1).

Like T-bet, Blimp-1 promotes the differentiation of CD8^+^  T_EFF_ while repressing transition into a central memory phenotype [137]. In a model of acute viral infection, Blimp-1 was shown to repress the expression of PD-1 both directly and indirectly by interfering with NFAT binding to the PD-1 promoter [138]. NFAT regulates the expression of PD-1 and Tim-3 and thus may contribute to CD8^+^ T cell exhaustion in chronic viral infection and cancer [139]. As mentioned earlier, disproportionate NFAT signaling is implicated in the induction of CD8^+^ T cell anergy, offering a potential role for this transcription factor in promoting more than one state of CD8^+^ T cell dysfunction in antitumor responses [140]. Blimp-1 may therefore prevent T cell dysfunction in early activated T cells through repression of both PD-1 and NFAT. In line with this idea, Blimp-1 was identified as a key regulator of CD8^+^ TIL effector function in advanced lung cancer patients. Blocking of miR-23a correlated with upregulation of Blimp-1, reacquisition of effector function, and delayed tumor progression [141]. The role of Blimp-1 in CD8^+^ T cells during chronic viral infection differs greatly from a well-controlled infectious challenge. PD-1^hi^ CD8^+^ T cells had 2 to 3 times more Blimp-1 expression than PD-1^int/lo^ CD8^+^ T cells. Similarly, iR^hi^ (PD-1, LAG-3, 2B4, and CD160) cells all had higher levels of Blimp-1 expression as compared to iR^lo^ CD8^+^ T cells. Blimp-1 expression correlated with a higher number of coexpressed iRs on a per cell basis. Importantly, conditional deletion of Blimp-1 was unable to rescue CD8^+^  T_EX_ because Blimp-1 induces granzyme B expression and cytotoxic activity [142]. Thus, Blimp-1 is important in promoting critical effector functions in acute immune responses but correlates with markers of CD8^+^ T cell exhaustion in chronic viral infection and possibly cancer.

Recent studies have implicated basic leucine zipper transcription factor (BATF) in CD8^+^ T cell exhaustion. BATF was shown to drive T-bet and Blimp-1 expression while inhibiting granzyme B and IFN*γ* in early effector CD8^+^ T cells. Thus, BATF promotes expression of transcription factors involved in effector differentiation but prevents effector molecule expression, suggesting that BATF may impede progression to an exhausted phenotype [143]. However, PD-1 expression was found to upregulate expression of BATF in HIV-specific CD8^+^ T cells, which inhibited T cell function. Signaling through PD-1 upregulated BATF expression, which in turn decreased T cell effector function through reduced proliferation and IL-2 production [144]. Thus, iRs may suppress CD8^+^ T cell-mediated antitumor immunity twofold, through diminished TCR signaling as well as regulation of context-specific transcription factors that influence CD8^+^ T cell fate.

TGF-*β* is an immunosuppressive cytokine that is released by CD4^+^  T_regs_ and APCs in the tumor microenvironment and directly inhibits CTL-mediated antitumor immune responses [119, 145–148]. Inhibition of CD8^+^ T cell function involves the formation of Smad (mothers against decapentaplegic homolog) transcription factor complexes. High-affinity DNA-binding is achieved by Smad interaction with coregulatory molecules such as FoxP1 (forkhead box). FoxP1 is upregulated in CD8^+^ TIL in the tumor microenvironment and necessary for TGF-*β*-mediated suppression of TIL, preventing rejection of ovarian tumors* in vivo* [149, 150]. Recent studies suggest that there may also be an antitumor effect of TGF-*β* signaling in CTL-mediated antitumor immunity. The TGF-*β* downstream molecules Smad2/3 and NFAT-1 were shown to promote CD103 expression on CD8^+^ TIL, an integrin that binds E-cadherin on tumor cells and induces cell lysis through granule exocytosis [151, 152]. In a separate study, TGF-*β* was shown to repress KLRG1 expression in CD8^+^ T cells* in vitro*. KLRG1 is an iR specific for E-cadherin and therefore inhibits CTL-mediated responses against E-cadherin expressing cells. TGF-*β*-deficient CD8^+^ T cells exhibited higher KLRG1 expression* in vivo*, suggesting that TGF-*β* may promote CTL-mediated tumor rejection through reciprocal regulation of KLRG1 and CD103 [153] (Figure 1). In line with this idea, Quatromoni et al. demonstrated that early blockade of TGF-*β* signaling prevented expansion of CD8^+^ TIL and negatively correlated with tumor volume, implying that some level of TGF-*β* signaling may be critical in generating CTL-mediated tumor rejection [154]. Conflicting evidence concerning the role of TGF-*β* signaling on CD8^+^ TILs highlights the need for more in-depth investigation. Studies have demonstrated both antitumor and protumorigenic roles for Smad transcription factors [155–158]. Therapies that aim to block TGF-*β* signaling in the tumor microenvironment are of high interest and have generated favorable responses in clinical trials, yet the importance of TGF-*β* signaling on CD8^+^ TIL in the tumor microenvironment remains to be determined [159, 160].

## 5. Conclusion

One of the current foci in the field of immunology is delineating the function of the adaptive immune system in antitumor responses. While cytotoxic CD8^+^ T lymphocytes are capable of recognizing and directly lysing transformed cells, CD8^+^ tumor-infiltrating lymphocytes often display dysfunctional properties* in vivo*. Reasons for CD8^+^ T cell impairment remain incompletely understood, but recent studies have identified multiple states of CD8^+^ T cell dysfunction in cancer patients as well as experimental models. These subsets include exhausted, anergic/tolerant, and regulatory/senescent CD8^+^ T cells. The current characterization of these dynamic fates in terms of surface marker profile and transcription factor expression is not sufficient to clearly delineate distinct CD8^+^ T cell fates. Transcription factors and inhibitory receptors exhibit multiple levels of crosstalk and feedback signaling both in early activated T_EFF_ cells and in the context of persistent antigenic stimulation, leading to diverse CD8^+^ T cell fates. Many of the key transcription factors that promote an effector phenotype also promote iR expression, perhaps maintaining an equilibrium between effector function and autoimmunity. In the context of antitumor immunity, increased iR expression limits CTL-mediated tumor rejection by promoting CD8^+^ T cell dysfunction. Novel immunotherapies that target multiple iRs may reverse the transcriptional network that regulates CD8^+^ T cell dysfunction and promote the adoption of effector and memory fates associated with active antitumor immunity.

## Figures and Tables

**Table 1:** Classification of human CD8^+^ T cell fates based on surface markers, transcription profiles, and observed phenotype.

CD8^+^ T cell fate	Surface marker profile	Transcription profile	Phenotype
Effector [18–22]	(i) KLRG1^+^ (ii) CD43^+^ (iii) CD62L^−^ (iv) CD69^+^ (v) CD95^+^ (vi) CD137^+^	(i) T-bet^hi^/Eomes^hi^ (ii) Blimp-1 (iii) Runx3 (iv) Stat4/Stat5 (v) Id2	(i) Direct cytotoxicity against transformed and virus-infected cells (ii) Mediate cytotoxicity through Fas/FasL and granzyme/perforin

Central memory [23–28]	(i) CCR7^+^ (ii) CD44^+^ (iii) CD45RO^+^ (iv) CD62L^+^ (v) CD122^+^ (vi) CD127^+^ (vii) IL15R^+^	(i) T-bet^lo^/Eomes^hi^ (ii) Bcl6 (iii) Tcf1 (iv) Stat3 (v) Id3 (vi) WNT-*β*-catenin	(i) Less differentiated (ii) Residing in lymph nodes, spleen, bone marrow, and blood (iii) No immediate effector function (iv) Differentiating into T_EFF_ upon antigen rechallenge (v) Self-renewal capacity (vi) IL-7/IL-15 dependence

Effector memory [23–28]	(i) CCR7^−^ (ii) CD44^+^ (iii) CD45RO^+^ (iv) CD62L^−^ (v) CD127^+^ (vi) KLRG1^+^	(i) T-bet^int^/Eomes^int^ (ii) Blimp-1/Bcl-6	(i) Found in both lymphoid and peripheral tissues (ii) Rapidly release effector molecules (iii) Highly cytotoxic (iv) Intermediate differentiation stage (v) Rapidly differentiate into T_EFF_ upon antigen rechallenge

Exhausted [29–33]	(i) CD45RO^+^ (ii) CD57^+^ (iii) CD95^+^ (iv) PD-1^+^ (v) CTLA-4^+^ (vi) Tim-3^+^ (vii) Lag-3^+^ (viii) BTLA^+^	(i) NFAT (ii) T-bet^lo^/Eomes^hi^ (iii) Blimp-1 (iv) BATF (v) FoxP1	(i) Reduced proliferation (ii) Decreased cytokine production (iii) Reduced cytotoxicity (iv) Reduced IFN*γ* and IL-2 secretion (v) Eventual cell death

Anergic/tolerant [34–41]	(i) Lag-3^+^ (ii) PD-1^+^	(i) NFAT (ii) NF-kB/RelA (iii) Ikaros (iv) Egr1/Egr2	(i) Reduced IL-2 secretion (ii) Reduced proliferation

Senescent/regulatory [42–44]	(i) KLRG1^+^ (ii) CD28^−^ (iii) CD57^+^	(i) FoxP3	(i) Cell-cycle arrest (ii) Immunosuppressive
